# Knowledge and attitudes of health care workers about monkeypox virus infection in Southern Italy

**DOI:** 10.3389/fpubh.2023.1091267

**Published:** 2023-02-27

**Authors:** Grazia Miraglia del Giudice, Giorgia Della Polla, Lucio Folcarelli, Annalisa Napoli, Italo Francesco Angelillo, The Collaborative Working Group, Walter Longanella

**Affiliations:** ^1^Department of Experimental Medicine, University of Campania “Luigi Vanvitelli”, Naples, Italy; ^2^Department of Public Health and Laboratory Services, Teaching Hospital of the University of Campania “Luigi Vanvitelli”, Naples, Italy

**Keywords:** attitude, health care worker, Italy, knowledge, monkeypox

## Abstract

**Background:**

This present survey sought to investigate the level of knowledge and the attitudes pertaining the monkeypox (mpox) virus infection among a sample of health care workers (HCWs) in Italy, as well as the possible role of different factors on these outcomes.

**Methods:**

The cross-sectional survey was performed from July through October, 2022 at four randomly selected hospitals located in Southern Italy.

**Results:**

The questionnaire was completed by 421 HCWs, for an overall 59% response rate. Less than two-thirds were able to define the disease and the correct answer of the transmission mechanisms ranged from 22.8% for contact with contaminated objects to 75.8% through close contact with body fluids. Only 4% and 12.8% indicated HCWs and elderly/frail/people with underlying immune deficiencies as risk groups. The mean overall score of the knowledge assessment on mpox was 3.4 (0–9). The multivariate logistic regression analysis showed that HCWs with a lower number of years of working experience and those who had acquired information about mpox from scientific journals were more likely to have a higher level of knowledge. The average score of the perception of the severity of the disease was 6.3. A similar score with a value of 6.1 has been observed for the statement that mpox is a serious problem for the population. Regarding the level of concern about contracting mpox, the mean score was 5.1. Only 10.5% reported that they feel that this disease can be prevented, with an overall mean score of 6.5. Almost all HCWs reported that they are still living as usual, with no modification of their behavior for fear of contracting the mpox. The results of the multivariate logistic regression model showed that women, HCWs with a higher level of knowledge about mpox, and those who needed additional information about mpox were more likely to have a higher level of perception of the severity of the disease.

**Conclusion:**

This survey has demonstrated that HCWs had an unsatisfactory level of knowledge toward mpox and only nearly half showed positive attitudes. Strategic health training programs should be made so that knowledge can be acquired.

## 1. Introduction

Monkeypox (mpox) is a viral zoonosis caused by an enveloped double-stranded DNA virus that belongs to the Orthopoxvirus genus and the Poxviridae family (1–4) and intimate contact with an infected person, infectious rashes or lesions, body fluids, respiratory droplets, and sexual contact are mechanisms of transmission among humans (5, 6). The first identified known human case was recorded in 1970 in the Democratic Republic of the Congo and the cases primarily occur in the tropical rainforest regions of Central and West African areas (7). However, since early May 2022, an emerging broad outbreak of mpox infection is spreading in different geographical areas where the disease is not endemic, including Europe, Americas, Australia, and Middle East. The primary cause of the development and spread of this kind of epidemic disease is the interaction between humans and animals (8). As of January 9, 2023, more than 84.000 confirmed cases have been reported in more than 110 countries worldwide (9). In Italy, the first case of mpox was reported on May 20, 2022, and since the start of the outbreak and so far, as of January 10, 2023, a total of 951 confirmed cases have been reported (10). Moreover, on July 23, 2022, the World Health Organization declared the escalating current global mpox outbreak a Public Health Emergency of International Concern (11).

Taking into consideration the current mpox scenario, health care workers (HCWs) in their practice may play an active role in making effective and targeted strategies for the prevention of the disease by educating and influencing the population. Indeed, previous epidemiologic studies have clearly established that HCWs' level of knowledge and their communication toward the prevention of several infectious diseases are key strategies in motivations different groups of individuals (12–18).

Few studies have focused the attention on the knowledge and attitudes about the mpox (19–25) notably among HCWs (26–29). Identifying and understanding the knowledge and attitudes in this group is essential for the development of effective and strategic health communication. Therefore, this present survey sought to investigate the level of knowledge and the attitudes pertaining the mpox virus infection among a sample of HCWs in Italy as well as to understand the possible role of different factors on these outcomes.

## 2. Materials and methods

This survey is part of a larger research project also examining attitudes and practices about COVID-19 among HCWs. The methodology is described in greater detail in a previous manuscript (30) and briefly summarized below.

### 2.1. Setting and sampling

The cross-sectional survey was performed between July 28 and October 14, 2022 at four randomly selected hospitals located in the Campania region, Southern Italy. A total of 421 HCWs were randomly selected. The required sample size of 384 HCWs was determined assuming a frequency of 50% of respondents who had a high perception of the severity of mpox, with a two-sided 95% confidence interval, and a margin error of 5%.

### 2.2. Data collection

The health director of each hospital received an invitation letter for asking the permission to conduct the study in their institution that included detailed information about the study regarding the background, objectives, and methodology. After their permission, the research team identified an HCW in each ward who distributed the questionnaire to the randomly selected study participants, then collected the filled questionnaires within an envelope to maintain anonymity, and then returned directly to the research team. At the beginning of the questionnaire, it was specified the study objectives and procedure, the voluntary participation, that the respondents' identities remain anonymous to the research team, that they had the right to quit their participation at any stage without any restriction, and that filling and returning the questionnaire were considered as their consent to participate and agreement to the terms of the study. Participants did not receive any gift or financial incentive in appreciation for completing the survey.

### 2.3. Survey development

The average time needed to complete the self-administered survey was ~5 min and it was structured into four subsets of questions, each with a specific focus: 1. socio-demographic, general, and professional characteristics (14 questions), including gender, age, relationship status, degree of education, duration of employment in the health care profession, and area of working activity; 2. source(s) for searching their information about the mpox and the need for additional information (2 questions); 3. knowledge about mpox which contained six questions with topics such as the definition, cause, modes of transmission, risk groups, number of observed cases in Italy and in the geographic area. Five questions were open-ended, and one was multiple-choice; 4. mpox attitudes (4 questions) and behavior (1 question), measuring perception of seriousness and danger of the disease and importance of its prevention, perception of risk of getting the disease for themselves, for familiars, and colleagues, and whether they made any modification of their behavior for fear of contracting the mpox. The questions on the attitudes were rated on a Likert-type scale ranging from 1 to 10, where the maximum score of “10” was assigned for the most acceptable/desired attitude and “1” for the least desirable, while the question on the behavior had “yes” or “no” as response options. The survey was pilot tested for clarity and understandability on a convenience sample of 10 HCWs and none of them have been included in the study. Internal consistency was satisfactory, with a Cronbach's alpha coefficient of 0.77.

### 2.4. Statistical analysis

The statistical software STATA 15.1 was used to analyze the data. Frequency, mean, and standard deviation were used to describe the principal characteristics of the participants, as well as behavior and attitude toward mpox. Univariate analysis, by using chi-square test and Student *t*-test, was performed to evaluate predictors of the different outcomes of interest. Any independent variable with a *p*-value <0.25 in the univariate analysis was further included in the multivariate logistic regression models, where odds ratios (OR) and their corresponding 95% confidence intervals (CI) were calculated. It has been investigated whether several independent variables predicted the following primary research questions: level of knowledge about mpox (Model 1) and perception of the severity of the mpox (Model 2). A knowledge-based score was created for each participant by assigning 1 point for each correct answer regarding definition, cause, modes of transmission, and risk groups and 0 for each incorrect or no answer. The total score was calculated for everyone by adding the points of each of the 13 questions (maximum score 13). For the purpose of analysis, the outcome of Model 1 has been dichotomized according to the total knowledge score calculated for each individual, with the study sample that has been divided into two categories with cut-off point the median value of the score of 3 (< 3 = 0 and >3 = 1). The perception of the severity of the disease as dichotomized outcome of Model 2, with cut-off point the median value of 6 (score < 6 = 0 and >6 = 1). The following independent variables of interest were tested in the univariate analysis because they are potentially related to all outcomes: gender (male = 0; female = 1), age, in years (continuous), marital status (unmarried/separated/divorced/widowed = 0; married/cohabitant = 1), physician/dentist (no = 0; yes = 1), currently working in medical wards (no = 0; yes = 1), length of practice, in years (continuous), having underlying at least one chronic medical condition (no = 0; yes = 1), scientific journals as source of information about mpox (no = 0; yes = 1), and need for additional information on mpox (no = 0; yes = 1). The variable level of knowledge about mpox (< 3 = 0; ≥3 = 1) was also included in Model 2. Statistical significance was assessed by two-tailed tests with *p*-value equal or < 0.05.

## 3. Results

A total of 714 HCWs were randomly selected and invited to participate in the present survey, and 421 returned the questionnaire, for an overall response rate of 59%. The distribution of the main socio-demographic, general, and professional characteristics of the sample is summarized in Table 1. Most respondents were female, the average age was 41.7 years, less than half were married/cohabitant, more than half were nurses/midwives, more than two-thirds worked in medical wards, almost one-third had worked in a COVID-19 area, the mean length of working experience was 13.5 years, and only 15.9% reported at least one chronic medical condition.

**Table 1:** Main socio-demographic, general, and professional characteristics of the sample.

**Characteristics**	**N**	**%**
Age, years	41.7 ± 12.5 (22–77)^*^
**Gender**		
Female	273	65.5
Male	144	34.5
**Marital status**		
Unmarried/separated/divorced/widowed	194	53.3
Married/cohabited with a partner	221	46.7
**Professional role**		
Nurse/Midwife	225	53.4
Physician/Dentist	133	31.6
Other	63	15
Length of practice, years	13.5 ± 12 (1–44)^*^
**Current working area**		
Medical	295	70.4
Other	124	29.6
**Having worked in a COVID-19 area**		
No	285	67.7
Yes	136	32.3
**At least one chronic medical condition**		
No	354	84.1
Yes	67	15.9

Number for each item may not add up to total number of study population due to missing value.

^*^Mean ± Standard deviation (range).

**Figure 1:**
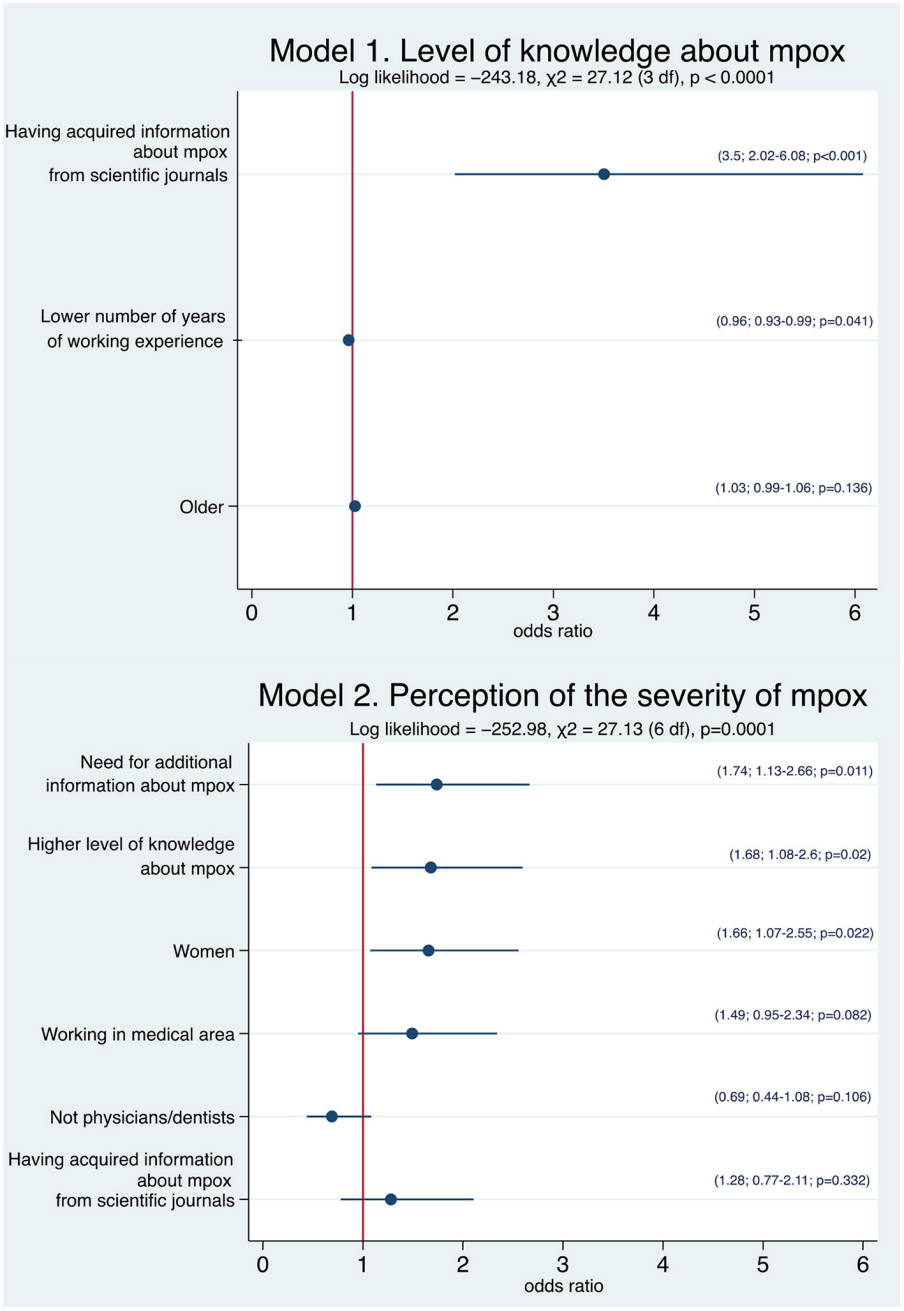
Forest plot of the associations of the several explanatory variables and the different outcomes of interest using multivariate logistic regression analysis. Circles represent the odds ratio for each individual variable and horizontal lines indicate 95% confidence interval. In brackets are reported odds ratio, 95% confidence interval, and *p-*value.

Table 2 showed the frequency of correct responses to each of the questions assessing mpox knowledge in the questionnaire. The overall level of knowledge was limited. No HCW gave the correct answer regarding all questions and 3.5% acknowledged that they do not know any of the answer. Regarding each question, 61.5% was able to define the disease and the correct answer of the transmission mechanisms ranged from 22.8% for contact with contaminated objects to 75.8% through close contact with body fluids. The knowledge of the participants referring to the risk groups, ranged from only 4% for those who indicated HCWs/laboratory personnel to 12.8% for elderly, frail, and people with underlying immune deficiencies. The mean overall score of the knowledge assessment on mpox was 3.4, with a minimum score of 0 and the maximum score of 9. The median value of the total score was found to be 3 and almost two-thirds (63.2%) had a score higher or equal than this value. The different factors associated with the two outcomes of interest on multivariate logistic regression analysis are reported in Figure 1. The results showed that the number of years of working activity and the sources of information about mpox yielded a statistically significant association with the knowledge level. HCWs with a lower number of years of working experience (OR = 0.96, 95% CI: 0.93–0.99) and those who had acquired information about mpox from scientific journals (OR = 3.5, 95% CI: 2.02–6.08) were more likely to have a higher level of knowledge than HCWs with a higher number of years and those who did not have used this source of information (Model 1).

**Table 2:** Frequency of correct responses to the questions assessing mpox knowledge.

**Items**	**Correct response**	**N**	**%**
1. What is mpox?	A viral infection	259	61.5
2. What is mpox caused by?	Mpox virus/poxvirus	106	25.2
3. How does mpox spread?	Close contact with body fluids	319	75.8
	Sexual intercourse	197	46.8
	Droplets	178	42.3
	Close contact with blood	122	29
	Contact with contaminated objects	96	22.8
4. Who is at risk of contracting mpox?	Frail people/elderly/people with underlying immune deficiencies	54	12.8
	Those who have unprotected sex/multiple sexual partners	20	4.7
	Those who have close contact with infected animals	19	4.5
	Men who have sex with men	18	4.3
	Those who have close contact with infected people	18	4.3
	HCWs/laboratory personnel	17	4

Participants' response to questions concerning their attitudes toward mpox, measured on a Likert-type scale ranging from 1 to 10, showed that the average score of their perception of the severity of the disease was 6.3 with 7.7% and 2.4% of participants selecting the “10” or “1” response, respectively. A similar score with a value of 6.1 has been observed for the statement that mpox is a serious problem for the population. When asked about their level of concern about contracting mpox, the mean score was 5.1 and 4.3% and 8.1% of all respondents said that they were very afraid of getting the mpox and not afraid at all, respectively. Additionally, only 10.5% respondents reported that they feel that this disease can be prevented and the overall mean score toward this attitude was 6.5. Finally, almost all HCWs reported that they are still living as usual, with no modification of their behavior for fear of contracting the mpox. The results of the multivariate logistic regression model showed that three variables reached statistically significant association with HCWs' perception of the severity of the disease. Women (OR = 1.66; 95% CI: 1.07–2.55), HCWs who had a higher level of knowledge about mpox (OR = 1.68; 95% CI: 1.08–2.6), and those who needed additional information about mpox (OR = 1.74; 95% CI: 1.13–2.66) were more likely to have a higher level of perception of the severity of the disease (Model 2 in Figure 1).

Lastly, the vast majority of the interviewed HCWs (89.6%) reported that they had search different sources to get information about mpox. Participants declared that their most trusted sources for obtaining information about this topic were the mass-media, Internet, and scientific journals, with values of 59.7%, 56.5%, and 28.6%, respectively. Almost two-thirds (64.6%) of all HCWs reported that that they would be interested in opportunities to learn more regarding mpox.

## 4. Discussion

This survey contributes to the limited scientific literature with new information regarding the level of knowledge and the attitudes pertaining the mpox virus infection as well as the contribution of factors that are associated among a sample of HCWs in the hospital settings in Italy. Numerous interesting themes emerged from the responses. Firstly, HCWs had a low level of knowledge toward mpox. Secondly, respondents had positive attitudes toward mpox. Thirdly, the vast majority reported getting information about mpox although only less than one-third from scientific journals. Fourthly, several factors have been observed to be associated with the two outcomes of interest.

This study revealed important knowledge gaps pertaining to mpox across the sample with a very low mean overall score, 3.4 out of 13, and this is also evident from the low number of HCWs who gave correct answers to the different questions, even about some basic aspects, related to definition, transmission mechanisms, and categories of people that are at higher risk. One possible explanation is that the frequency of the disease had not already achieved tremendous prominence at the time of this survey, and no activities had been made to raise the knowledge levels among HCWs, so they may not have reached an adequate level of knowledge. It is worth mentioning that the rate of correct response that a virus was the cause of the mpox (61.5%) in this study was consistent with the 61.9% observed in the Kingdom of Saudi Arabia among HCWs (31), whereas it was much lower than what has been reported elsewhere in previous studies, although some of them conducted among groups of individuals in different settings. Indeed, in two studies conducted in Jordan based on medical students and HCWs revealed values of 77.2% (22) and 92.6% (22), in China in the general population of 83.1% (32), in Italy in occupational physicians, public health professionals, and general practitioners of 95.1% (29), and in Kuwait in physicians of 99% (27). Moreover, the value was higher than that observed in Turkey among physicians with only 0.4% that were aware that this was a bacterial infection (33). Regarding the transmission mechanisms, the present study showed that the correct answers of the responding HCWs ranged from 22.8% for contact with contaminated objects to 75.8% through close contact with body fluids and 42.3% indicated the droplets. Some of the already mentioned studies have shown that the knowledge on the transmission through droplets were very similar to the present value. Indeed, 41.3% of HCWs in the Czech Republic (34), 45.3% in China (32), and 47.3% in Turkey (33) were aware of this mode of transmission, while 47.5% has been observed among Chinese men who have sex with men (35). Lastly, almost all (98.8%) Italian physicians acknowledged the potential transmission by means of respiratory droplets (29). In the present study, of concern was the knowledge gap about the groups at risk which ranged from 4% for those who indicated HCWs/laboratory personnel to 12.8% for elderly, frail, and people with underlying immune deficiencies. A considerably higher value has been observed in the previously cited survey among men who have sex with men with 40.2% of the respondents that correctly indicated them as high-risk group (35). The lack of knowledge of the disease, how it is transmitted as well as regarding risk groups is troubling because knowing is a prerequisite to facilitate the HCWs for an effective implementation of a successful control and educational activities and, therefore, this may have negative effects upon control and prevention efforts. Indeed, a body of literature showed that HCWs' knowledge is one of the most influential predictive factors regarding discuss and recommend primary preventive interventions to their patients (15, 36, 37). Therefore, based on the knowledge gaps pertaining to the different aspects of mpox, it is necessary and crucial for health authorities to encourage HCWs to obtain information from trustworthy sources and proper education and training are also needed to address misconceptions and to improve the level of knowledge. Additionally, it is interesting to underline that HCWs' knowledge toward mpox significantly affected their attitude. Indeed, HCWs who had a higher level of knowledge were more likely to have a higher level of perception of the severity of mpox.

Apart from identifying HCWs' knowledge, another purpose of this survey was to find their beliefs and attitudes toward mpox. An interesting result was that 4.3% felt that they were at high risk of getting mpox with an overall mean score of 5.1, measured on a Likert-type scale ranging from 1 to 10. The present finding does not allow inferring the basis on which this belief emerged, but we may speculate that it might have arisen from the fact that only a small number of respondents correctly indicated themselves as a group at risk. A previous study showed that 49.6% of HCWs in Saudi Arabia was afraid of contracting mpox (28) and in the already mentioned survey in Turkey 20.1% of participants were more concerned about mpox than COVID-19 (33), whereas 75% of men who have sex with men showed concerns about their susceptibility to mpox infection (35).

It should be noted that the most frequently cited sources of information about mpox were mass-media, Internet sites, and scientific journals. The observation of the high frequency of internet users for seeking information on this topic among respondents raises concern and this could perhaps partially explain the low level of knowledge since these sources have long been acknowledged in prior international literature to disseminate misleading health information (38–43). The multivariate logistic regression analysis found a positive association between HCWs' knowledge score and source of information. Indeed, participants who were exposed to scientific journals had greater odds of having a higher level of knowledge compared to those who did not have used it. This finding is not unexpected in view of the fact that HCWs can benefit from updated access to accurate and correct information and this is also supported by evidence which identified these sources for enhancing public health education on a variety of topics. Indeed, previously published literature have shown that individuals gathering information from scientific journals or from institutional sources had a higher level of knowledge, a more positive attitude, and were more likely to adopt appropriate public health behavior and to accept the vaccination (44–48). The finding here clearly illustrates how important the information is when it comes from scientific journals that, therefore, should be used more prominently as a regular source of information for HCWs as an important strategy for improving their level of knowledge. Lastly, another feature observed in the current survey that should be noted was the significant association between attitudes and information with those who reported that that they would be interested in acquiring more information about mpox were more likely to have a higher perceived level of the severity of the disease. Therefore, HCWs should be the target group for educational programs.

The multivariate logistic regression analysis in the current survey identified additional determinants as having a significant influence on the different outcomes of interest. Number of years of working activity in healthcare profession and gender were predictive of knowledge and attitude among the sampled HCWs. HCWs in activity with a lower number of years were more knowledgeable than those with more experience. One of the possible justifications might be that HCWs who have less experience are more active and might have more interest in acquiring information, read scientific journals, and participate in recent proper training and education than those with more years of activity. In addition, female HCWs were more likely to have a higher level of perception that the mpox is characterized as a serious health risk, which is consistent with previous studies, in which female is an important factor for concern toward infectious diseases (44, 45, 49, 50).

There are inherent potential methodological limitations that should be considered when interpreting the results of the present survey. First, the cross-sectional design was designed to measure association between the explanatory variables and the different outcomes of interest, and a causal relationship cannot be determined. Second, the participants were selected from hospitals located only in one region of the country. Therefore, there is a possibility that the study findings may not be entirely generalizable to the true level of knowledge and attitudes of other HCWs across the country. Third, a self-administered questionnaire had been used to collect data and participants may answer questions in a socially desirable manner so conclusions may contain social desirability bias. However, the questionnaire was anonymous with no identifying data collected and this may have reduced the risk of such bias. Despite the limitations, this data provides relevant and valuable information on the level of knowledge and attitudes and the associated factors of Italian HCWs toward mpox.

In conclusion, this unique survey has demonstrated that HCWs had an unsatisfactory level of knowledge toward mpox and nearly half showed positive attitudes. Gender, years of working activity, and sources of information were the significant determinants of knowledge and attitude levels, and these factors should be taken into account when tailoring effective and strategic health training programs should be made. Therefore, the recommendation considering the results is that such programs for HCWs should be made so that knowledge about the risks posed by the mpox, as well as by other zoonotic infectious diseases, and the preventive measures can be acquired.

## Data availability statement

The raw data supporting the conclusions of this article will be made available by the authors, without undue reservation.

## Ethics statement

Ethical review and approval were not required for the study on human participants in accordance with the national legislation. Informed consent from the participants was obtained by filling and returning the questionnaire.

## The collaborative working group

Walter Longanella (Health Direction, San Giovanni di Dio Ruggi D'Aragona Hospital, Largo Città Ippocrate, 84131 Salerno, Italy), Mario Massimo Mensorio (Health Direction, Sant'Anna e San Sebastiano Hospital, Via Ferdinando Palasciano, 81100 Caserta, Italy), Federica Cantore (Health Direction, San Giuseppe Moscati Hospital, Contrada Amoretta, 03100 Avellino, Italy).

## Author contributions

GMdG, GDP, LF, and AN participated in the conception and design of the study and contributed to the data collection. GMdG and GDP contributed to the data analysis and interpretation. IFA the principal investigator, designed the study, was responsible for the statistical analysis and interpretation, and wrote the article. All authors have read and approved the final version of the article and agree to be accountable for all aspects of the work.
